# From Microbial Ecology to Clinical Challenges: The Respiratory Microbiome’s Role in Antibiotic Resistance

**DOI:** 10.3390/pathogens14040355

**Published:** 2025-04-05

**Authors:** Adelina-Gabriela Niculescu, Mihaela Magdalena Mitache, Alexandru Mihai Grumezescu, Mariana Carmen Chifiriuc, Mara Madalina Mihai, Monica Marilena Tantu, Ana Catalina Tantu, Loredana Gabriela Popa, Georgiana Alexandra Grigore, Roxana-Elena Cristian, Mircea Ioan Popa, Corneliu Ovidiu Vrancianu

**Affiliations:** 1 Department of Science and Engineering of Oxide Materials and Nanomaterials, National University of Science and Technology POLITEHNICA Bucharest, 011061 Bucharest, Romania; adelina.niculescu@upb.ro (A.-G.N.); agrumezescu@upb.com (A.M.G.); 2 Research Institute of the University of Bucharest—ICUB, University of Bucharest, 050663 Bucharest, Romania; grigore.georgiana-alexandra@s.bio.unibuc.ro (G.A.G.);; 3 Department of Preclinical Disciplines, Faculty of Medicine, Titu Maiorescu University, 031593 Bucharest, Romania; magda.mitache@yahoo.com; 4 Microbiology-Immunology Department, Faculty of Biology, University of Bucharest, 050095 Bucharest, Romania; 5 Biological Sciences Division, Romanian Academy, Calea Victoriei 125, Sector 1, 010071 Bucharest, Romania; 6 Faculty of Medicine, “Carol Davila” University of Medicine and Pharmacy, 020021 Bucharest, Romania; mara.mihai@umfcd.ro; 7 Department of Oncologic Dermatology, “Elias” University Emergency Hospital, 010024 Bucharest, Romania; 8 Department of Medical Assistance and Physical Therapy, Pitesti University Center, Târgu din Vale 1, 110040 Pitești, Romania; tantumonica@yahoo.com; 9 Faculty of Science, Physical Education and Informatics, National University of Science and Technology, Politehnica, Splaiul Independenței 313, District 6, 060042 Bucharest, Romania; 10 Doctoral School, University of Medicine and Pharmacy of Craiova, Petru Rareș 2, 200349 Craiova, Romania; catalina.tantu8@gmail.com; 11 Emergency Clinical County Hospital of Craiova, Tabaci 1, 200642 Craiova, Romania; 12 Microbiology Discipline II, Faculty of Medicine, Carol Davila University of Medicine and Pharmacy, 020021 Bucharest, Romania; dr.gabriela.popa@gmail.com (L.G.P.); mircea.ioan.popa@gmail.com (M.I.P.); 13 National Institute of Research and Development for Biological Sciences, 296 Splaiul Independentei, District 6, 060031 Bucharest, Romania; 14 Preclinical Testing Unit, Cantacuzino National Military Medical Institute for Research and Development, 050096 Bucharest, Romania; 15 Doctoral School, Carol Davila University of Medicine and Pharmacy, Eroii Sanitari 8, District 5, 050474 Bucharest, Romania

**Keywords:** respiratory microbiome, antibiotic resistance, respiratory pathogens, alternative antimicrobials, gut–lung axis, microbial restoration, precision medicine

## Abstract

Antibiotic resistance represents a growing public health threat, with airborne drug-resistant strains being especially alarming due to their ease of transmission and association with severe respiratory infections. The respiratory microbiome plays a pivotal role in maintaining respiratory health, influencing the dynamics of antibiotic resistance among airborne pathogenic microorganisms. In this context, this review proposes the exploration of the complex interplay between the respiratory microbiota and antimicrobial resistance, highlighting the implications of microbiome diversity in health and disease. Moreover, strategies to mitigate antibiotic resistance, including stewardship programs, alternatives to traditional antibiotics, probiotics, microbiota restoration techniques, and nanotechnology-based therapeutic interventions, are critically presented, setting an updated framework of current management options. Therefore, through a better understanding of respiratory microbiome roles in antibiotic resistance, alongside emerging therapeutic strategies, this paper aims to shed light on how the global health challenges posed by multi-drug-resistant pathogens can be addressed.

## 1. Introduction

The increasing rates of antibiotic resistance represent an escalating threat to public health, mainly due to pressing consequences such as heightened mortality rates and prolonged illnesses [1,2]. This issue is particularly alarming for airborne pathogens harboring acquired drug resistance, which can be transmitted easily and cause respiratory infections in all populations, thus becoming a global healthcare priority [2,3,4,5]. The significance of airborne transmission in disseminating ARGs in the environment is often underestimated despite its pivotal role. Therefore, there is an urgent need to understand the mechanisms, origins, and distribution of antibiotic resistance for effective short- and medium-term management of airway infections [3,6].

Antibiotic resistance research has primarily focused on the gut microbiome, with comparatively less attention given to the respiratory tract resistome [4]. Recent developments in culture-independent methods have enhanced scientists’ capacity to characterize the variety of microbial species that inhabit particular host sites, the changes in composition and function that occur during disease states, and the specific community members that are strongly associated with the severity of clinical symptoms or with triggering specific immune responses [7]. Despite this, ARGs’ diversity and abundance in the human RTM are still poorly understood [4].

Moreover, in spite of certain improvements, acute and chronic lower airway illnesses continue to significantly contribute to worldwide morbidity and mortality. In addition, the economic impact of treating lower respiratory tract infections and productivity losses is considerable [8,9], being especially exacerbated by the emergence of multi-drug-resistant (MDR) bacteria and viral strains variations [10]. Thus, the expected rise in MDR infections without successful interventions highlights the urgent need for alternative preventative measures and therapies [2,4,5,11].

In this context, this review proposes a comprehensive path for better understanding the role of the RTM as a reservoir of antibiotic resistance. Starting from describing the RTM in health and disease and overviewing antibiotic resistance, this paper analyzes the interactions between the respiratory microbiome and antibiotic-resistant pathogens, further overviewing therapeutic strategies. Specifically, antibiotic stewardship programs, alternatives to conventional antibiotics, probiotics, and microbiome restoration methods are discussed as approaches to preserving respiratory health and combating antibiotic resistance.

Table 1 introduces the definitions of some of the most relevant terms to help readers better understand the following sections.

## 2. Respiratory Microbiome in Health and Disease

Spanning from the nostrils to the lung alveoli, the respiratory tract encompasses the suprathoracic (upper) respiratory tract (URT) and the intrathoracic (lower) respiratory tract (LRT), boasting a vast surface area of approximately 100 m^2^ that homes a microbial ecosystem known as the respiratory tract microbiome (RTM) [11]. Once considered sterile below the larynx, the tracheobronchial tree and lung parenchyma are now recognized to host diverse microbial species, spanning bacteria, fungi, and archaea as well as viruses, termed the “microbiome” [21,22,23]. The dynamics and complexity of RTM have been revealed by the molecular techniques’ advancements, allowing culture-independent lung explorations [24,25]. Within this ecosystem, the RTM thrives across various airway niches, fostering respiratory tract health and exerting a crucial influence on lung infection outcomes [7,26,27,28].

Even though the RTM is less studied than the microbial communities from other sites, it is an essential contributor to the host’s local immune behavior and respiratory disease development [29]. The composition of RTM is influenced by various factors, such as nutrient availability, local geography, and physicochemical conditions (e.g., mucociliary escalator, oxygen levels, blood flow, pH, temperature, interactions with the human immune system, and environmental factors) [11,30].

A balanced RTM plays a crucial role in respiratory health by preventing the colonization of respiratory pathogens, contributing to the immunological tolerance of the lung microenvironment, and strengthening the immune system [17,31]. This balance is maintained by a diverse bacterial composition, primarily consisting of bacteria from the phyla *Firmicutes*, *Bacteroidetes*, and *Proteobacteria*, among the most common phyla in all human site-specific microbiomes. Notably, *Streptococcus*, *Prevotella*, and *Veillonella* genera are dominant in the RTM and closely linked to the oral microbiota. Although bacteria constitute most of the RTM, several studies also identified other microbial members, such as fungi [32,33,34], archaea [35,36], or viruses [11,37]. Thus, the normal microbiota of the nasopharyngeal, tracheobronchial, and pulmonary sectors in healthy subjects is characterized by a low density and high diversity. Nonetheless, in pathological conditions, certain microorganisms increase while others decrease, leading to imbalances in the microbiome. If the microbiome is altered, pathogenic agents could thrive locally or spread to other locations, leading to various infections (e.g., acute rhinosinusitis, otitis media, pneumonia, and sepsis) [29].

**Figure 1:**
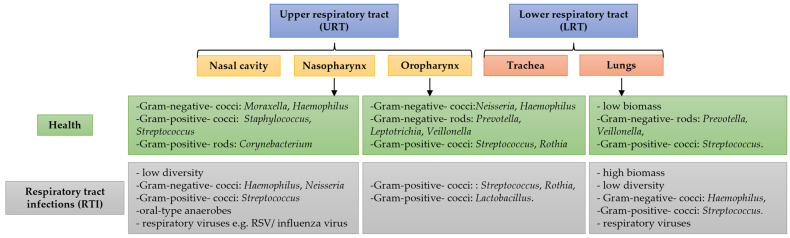
Main bacterial genera and microbiome species of an adult in the URT and LRT.

The healthy RTM in the URT supposes the colonization of mucous surfaces from the nasal cavity, rhino-pharynx, and oropharynx with various bacteria [29,38], as depicted in Figure 1. As healthy adults inhale over 7000 L of air daily, the URT is continuously exposed to environmental particles, including a substantial influx of bacteria.

Microbial exposure, coupled with various atmospheric factors (e.g., humidity and oxygen levels), immunological agents, and nutrients, contributes to bacterial density and various changes in the URT.

Several studies investigated the role of humidity and ventilation in bacterial growth and microbiota composition, suggesting that conditions below and above the optimal range could facilitate infectious transmission and exacerbate respiratory diseases [39]. Qiu et al. [40] showed that increased ventilation and reduced humidity could significantly reduce bacterial growth at 26 °C and 34 °C. In addition, high temperatures significantly increased bacterial growth, while ventilation had an opposite effect. Rasheed et al. [41] revealed a fivefold increase in *Pseudomonas aeruginosa* survival rate under low evaporation, highlighting the critical impact of environmental conditions on pathogen persistence and disease spread from inanimate surfaces. A study focusing on the effect of temperature and relative humidity on hospitalization for acute LRT infections in children demonstrated that both low and high relative humidity had a detrimental effect on hospitalization [42].

The investigation of oxygen levels in patients with cystic fibrosis demonstrated that hyperoxia reduced specific bacterial load, including facultative anaerobes such as *Rothia* and some *Streptococcus* species, with a slight decrease in *P. aeruginosa* and *Staphylococcus aureus* pathogens [43].

These factors may lead to disruptions in the microbial balance of the URT, which may further cause opportunistic pathogens colonization, eventually leading to more severe respiratory infections and diseases [44,45,46].

**Figure 2:**
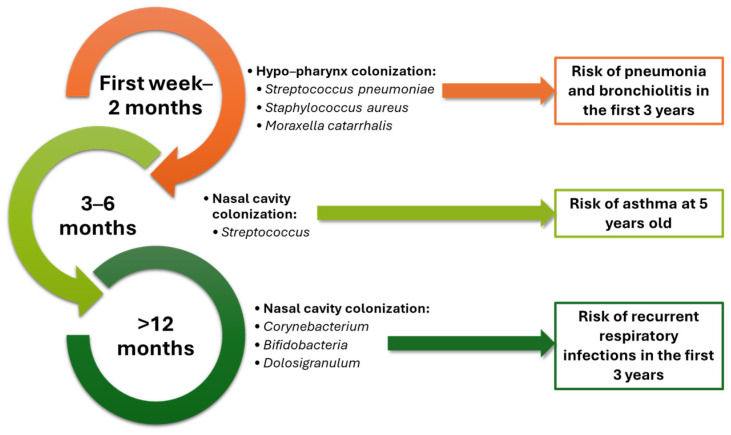
The evolution of the URT microbiome during human life and risk of respiratory tract diseases. Adapted from an open-access source [29].

Moreover, the URT microbiome undergoes significant changes throughout human life. Initial colonization is influenced by delivery mode, with major shifts in microbial composition occurring in the first year as the immune system matures (Figure 2). This early community gradually evolves into the diverse yet less dense adult URT microbiome [46]. A recent study showed that the presence of *Streptococcus pneumoniae* in the nasopharyngeal microbiota of adults leads to a shift in the microbiota, with the dominance of the *Streptococcus* genus. In addition, adults who regularly interact with children have a microbiota enriched in pathobionts frequently carried by children, including *Streptococcus* sp., *Moraxella catarrhalis*, and *Haemophilus influenzae* [47]. While early-life modulation of the infant URT microbiome might impact susceptibility to respiratory infections, further rigorous studies and regulatory improvements for bacterial products are necessary to advance this field [48].

**Figure 3:**
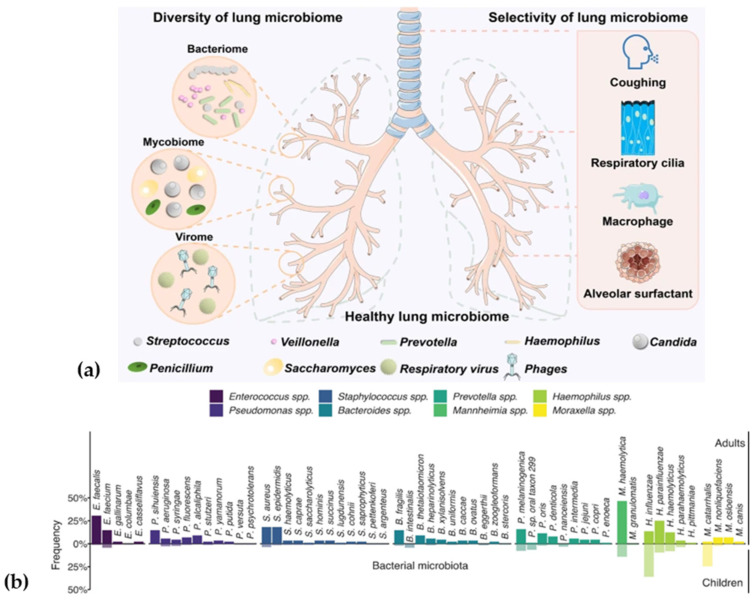
(**a**) The composition of the healthy lung microbiome, reprinted from an open-access source [52]. (**b**) Lung bacterial microbiome of children compared with adults. Frequency of the bacterial species detected in ≥5% of children (translucent) and adults (solid) among the differentially abundant bacterial genera. Reprinted from an open-access source [51].

In contrast, the LRT, including the trachea and lungs, has low microbial biomass, controlled by efficient microbial clearance mechanisms [49]. The lung microbiome is characterized by dynamic fluxes of microbial immigration and clearance, its composition dominated by bacteria from the phyla *Bacteroidetes*, *Actinobacteria*, and *Firmicutes*. Its total bacterial abundance increases with age, and community composition differs between children and adults. Several bacterial genera, such as *Enterococcus*, *Pseudomonas*, *Staphylococcus*, *Bacteroides*, *Prevotella*, *Mannheimia*, *Haemophilus*, and *Moraxella*, exhibit significant variations in prevalence and species composition across age groups, underscoring the dynamic and age-specific nature of the RTM [50,51] (Figure 3).

With the increasing understanding of the lung microbiome, it becomes evident that the disruption of the microbial–host interface profoundly influences disease pathogenesis. For instance, the airway microbiome significantly contributes to asthma pathogenesis, with noticeable changes in early development and established disease.

Several studies on the lower airway microbiome in early childhood revealed the association between alterations in microbial composition and the development of wheezing and asthma [49]. For example, two studies enrolling 80 hospitalized children with recurrent wheezing symptoms revealed significant changes in the lower airway microbiota, mainly in the *Proteobacteria*, *Bacteroidota*, *Fusobacteriota*, and *Acidobacteriota phyla*, with higher abundances of *Elizabethkingia* and *Rothia* and lower abundances of *Fusobacterium* [53,54]. In addition, there is a growing body of evidence that bacterial pathogens detected during acute wheeze episodes in the neonatal nasopharyngeal microbiome, mainly *S. pneumoniae*, *H. influenzae*, and *M. catarrhalis*, are associated with early childhood wheeze and the presence of asthma at the age of 5 years [55,56]. RMT of asthmatic adults comprises a less diverse bacterial community than healthy people and increased richness. Specifically, an overrepresentation of the *Proteobacteria* phylum has been linked to asthma exacerbations and the induction of Th17-related immune responses. In particular, *Haemophilus* and *Moraxella* genus enrichment further correlates with severe airway obstruction, neutrophilic inflammation, and poor asthma control [50]. Other changes noted in asthma patients include an increased abundance of pathogenic taxa, such as *Staphylococcus* and *Actinomyces*, and a concurrent reduction in commensals like *Prevotella* and *Veillonella*. In severe asthma, higher levels of *Pseudomonas* are often observed despite this pathogen being rarely detected in healthy lungs. In hospitalized patients with atopic asthma, the tracheal microbiome presents enhanced levels of *Haemophilus*, *Fusobacterium*, *Neisseriaceae*, *Sphingomonas*, and *Porphyromonas*. In contrast, the levels of *Bacteroides* and *Lactobacillus* are reduced compared to healthy microbiomes [52]. These findings underscore the complex relationship between microbial dysbiosis and asthma severity.

Several particularities have also been noted regarding chronic obstructive pulmonary disease (COPD), a severe respiratory condition with substantial global health and economic impacts. The lung microbiome’s importance in COPD progression has been increasingly recognized, particularly during acute exacerbations. Comparing the microbiome in COPD patients and healthy controls, studies have demonstrated altered microbial diversity, with increased relative abundance of *Moraxella*, *Streptococcus*, *Proteobacteria*, *Veillonella*, *Eubacterium*, and *Prevotella.* In severe COPD (GOLD stage 4), the most significant modifications assume an increase in *Proteobacteria* and a reduction in *Firmicutes*, *Bacteroidetes*, *Streptococcus*, *H. influenzae*, and *Prevotella*. These changes appear to be associated with disease progression and may be independent of smoking history, with microbial diversity varying across specific COPD endotypes [57].

On the other hand, the presence of potentially pathogenic microorganisms like *Haemophilus* spp., *S. pneumoniae*, *M. catarrhalis*, *S. aureus*, and *P. aeruginosa* in COPD patients has been correlated to neutrophilic inflammation and increased cytokine expression, including IL-8 and TNF [49]. Moreover, abundant opportunistic pathogens like *P. aeruginosa* and *Lactobacillus* cause the worsening of airflow restriction [52]. Altogether, these findings emphasize the dynamic and evolutionary nature of the lung microbiome and its pivotal role in COPD pathogenesis and progression.

In contrast to COPD, the cystic fibrosis (CF) lung microbiota exhibits a distinct trajectory characterized by significant inter-individual variability in structure, composition, and diversity. The core CF microbiota includes genera such as *Streptococcus*, *Prevotella*, *Rothia*, *Veillonella*, and *Actinomyces*. While CF-associated pathogens like *Pseudomonas*, *Burkholderia*, *Stenotrophomonas*, and *Achromobacter* are less prevalent than core genera, they often dominate the microbial community when present. Opposed to the non-CF lung microbiome (which shows increased diversity with age), the CF lung microbiome encounters a declining diversity over time. Community diversity and lung function are highest in patients under 10 years old and decline with age, plateauing by approximately age 25. The reduced microbial diversity has been strongly associated with worsening lung function, elevated levels of IL-8, and neutrophil elastase, highlighting the role of inflammation. Age-associated changes include increased abundance of *Pseudomonas* and *Staphylococcus*, alongside decreased prevalence of *Streptococcus*, *Porphyromonas*, and *Veillonella* [58,59]. These findings suggest a potential relationship between declining microbial diversity, pathogen dominance, and progressive respiratory dysfunction in CF patients.

Relevant changes have also been documented in bronchiectasis, a heterogeneous respiratory condition characterized by permanent airway enlargement, chronic cough, sputum production, and recurring infections of various etiologies. Non-CF bronchiectasis is characterized by persistent colonization with bacteria such as *P. aeruginosa* and *H. influenzae*, associated with frequent exacerbations, neutrophil-mediated inflammation, and reduced lung function. Microbial profiling also shows a reduction in diversity and dominance of some taxa, such as *Proteobacteria*. Conversely, *Firmicutes* like *Veillonella* correlate with frequent exacerbations despite milder inflammation. Fungi and nontuberculous mycobacteria (NTM) can additionally contribute significantly to disease progression. *Aspergillus* species are frequently encountered in diseased lungs and are associated with airway inflammation and exacerbations. NTM is recognized as both a cause and contributor to microbiome dysbiosis, further disrupting the microbial community through its influence on antimicrobial therapies [57].

### 2.1. Impact of Antibiotics on the Respiratory Microbiome

Despite their prior status as revolutionary treatments for infectious diseases, antibiotics are increasingly acknowledged for their profound effects on human health, including the disruption of the delicate microbial communities within the body. They contribute to the antimicrobial resistance (AMR) global health crisis driven by the evolution of bacterial pathogens in response to the selective pressures of antibiotic use in medicine and agriculture [60]. Epidemiological evidence underlines a cause-and-effect relationship between antibiotic overuse and the emergence of resistant strains, but despite this, inappropriate prescribing and consumption remain alarmingly widespread. In this context, the situation escalated near the “point of no return,” where traditional treatments are increasingly ineffective against resistant pathogens. Thus, AMR renders many infections difficult (if not impossible) to treat, being projected to cause up to 10 million deaths annually by 2050 if unaddressed [61,62,63,64].

The development of resistance mechanisms arises from both intrinsic and acquired factors. Intrinsic resistance accounts for the inherent physiological traits of certain bacteria, such as altered membrane structures, biofilm formation, or the absence of specific drug targets, which naturally limit antibiotic efficacy [65]. Such an example is *Mycobacterium tuberculosis*, which exhibits resistance through its thick, lipid-rich cell wall, granuloma formation that impedes antibiotic penetration, and a dormant state that decreases metabolic activity, enhancing resilience to antibiotics [63].

On the other hand, acquired resistance is determined by genetic changes, occurring either through spontaneous mutations or horizontal gene transfer (HGT) [3,65]. HGT is one of the main drivers of resistance spread, enabling bacteria to exchange resistance genes via different mechanisms, e.g., conjugation (direct DNA transfer), transformation (uptake of naked DNA), and transduction (bacteriophage-mediated transfer). Once acquired, these resistance traits are propagated within bacterial populations through vertical gene transfer. Key examples include β-lactamase enzymes (that inactivate penicillin and related drugs) and the mcr-1 gene (which confers resistance to colistin). These mechanisms easily and quickly spread worldwide, threatening even last-resort therapeutic options, rendering them ineffective [3,63,65].

**Figure 4:**
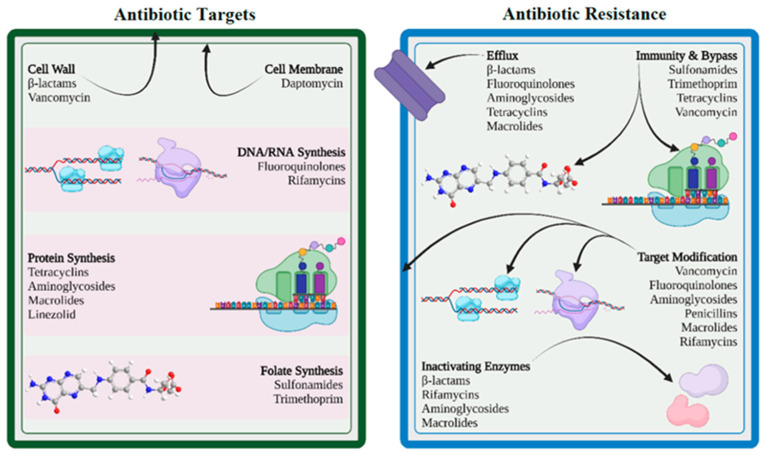
Schematic representation of antibiotic targets and resistance mechanisms. Reprinted from an open-access source [64].

A bacterium is considered resistant if its minimum inhibitory concentration (MIC) for an antibiotic surpasses the established breakpoint. This indicates that it is unlikely to effectively inhibit the isolate at the recommended dose for treating the specific infection [66]. Bacterial resistance strategies are diverse and highly effective, including the reduction in intracellular antibiotic concentrations through efflux pumps or decreased cell envelope permeability, alteration of drug targets via mutations or protective enzymes, direct inactivation of antibiotics through hydrolysis or modification, or bypassing metabolic pathways of the antibiotics target [64,65] (Figure 4).

The implications of AMR extend beyond genetic mechanisms, with MDR pathogens often displaying phenotypic adaptations that hinder therapy, such as biofilm-associated resistance or persister cells’ survival. For example, biofilms, as seen in *P. aeruginosa*, provide a physical barrier against antibiotics and foster phenotypic resistance through persister cell formation, often linked to chronic infections like those in cystic fibrosis patients [63].

Moreover, resistance frequently results in increased reliance on less effective, more toxic, or more expensive antibiotics, aggravating the public health burden. The rise in AMR is not confined to specific regions or pathogens, with resistance traits observed across numerous species, including *H. influenzae*, *S. pneumoniae*, *S. aureus*, and *Escherichia coli* [5,63,64,67,68].

Therefore, combating AMR necessitates immediate innovation in stewardship initiatives, surveillance, and treatment approaches. Targeting resistance mechanisms, disrupting biofilm structures, and developing alternative antimicrobial agents are critical for addressing AMR. Without concerted global efforts, the spread of AMR threatens to undermine decades of medical progress, elevating the risk of untreatable infections and worldwide health crises. The World Health Organization (WHO) has identified 12 antibiotic-resistant “priority pathogens, among which *P. aeruginosa*, *S. pneumoniae*, *H. influenzae*, and *S. aureus* are known to contribute to respiratory infections, particularly in individuals with underlying conditions such as immunosuppression, chronic lung diseases, cancer, recent surgery, or intensive care hospitalization [69].

Antibiotic-induced alterations to the microbiome are especially noticeable in the respiratory system. Even though the URT microbiota is often resilient, returning to its pre-antibiotic state relatively quickly, changes in the lung microbiome may be more persistent and harmful. Such disruptions can degrade microbial diversity, impair vaccine efficacy, and compromise the microbiome’s protective role against invasive infections. The balance between the beneficial and detrimental effects of antibiotics remains underexplored, especially in conditions like COPD, cystic fibrosis, and bronchiectasis, where frequent antibiotic use is both a necessity and a liability [69,70]. Addressing these issues requires a multifaceted approach prioritizing responsible antibiotic stewardship, additional in-depth research into microbiome dynamics, and innovative medicines that minimize collateral damage to microbial ecosystems.

Table 2 summarizes the essential findings on the influence of various antibiotics on the RTM throughout chronic respiratory illnesses. It sheds light on how antibiotic treatment affects microbial diversity and composition in disorders such as bronchiectasis, asthma, COPD, and CF. The table below offers an at-a-glance perspective on the differences in α- and β-diversity, changes in microbial taxa, and antibiotics’ impact on the respiratory microbiota across time. These findings underscore the complex interplay between antibiotic use and microbial communities within the airways, stressing antimicrobial interventions’ therapeutic benefits and side effects.

### 2.2. Antibiotic Resistance in Respiratory Pathogens

Antibiotic resistance among respiratory pathogens presents a significant challenge to public health, complicating the management of infections such as pneumonia, bronchitis, and tuberculosis. Infections caused by antibiotic-resistant pathogens lead to higher morbidity and mortality, also posing a considerable economic burden due to prolonged hospital stays and the necessity for more expensive therapeutic alternatives. Recent studies have highlighted the role of the lung microbiome as a reservoir for ARGs, which facilitate HGT among commensal and pathogenic bacteria, further exacerbating the spread of resistance within the respiratory tract [30,51,71,72,73].

The totality of airway ARGs is collectively known as the respiratory resistome [15,30]. The respiratory resistome represents a pivotal focus for comprehending antibiotic resistance mechanisms and their implications for treatment efficacy [30,66]. Molecular approaches, whether employed independently or alongside traditional in vitro susceptibility testing, have proven valuable in predicting the efficacy of antibiotic regimens by identifying specific ARGs strongly correlated with resistance, such as the mecA gene linked to methicillin-resistant *S. aureus* (MRSA) [66]. Especially in the context of chronic respiratory diseases (CRDs), advanced metagenomic analyses of the resistome have opened the door for a deeper understanding of antibiotic resistance dynamics, highlighting both core and disease-specific ARG repertoires [6,30,74].

The core airway resistome exists in both CRD (e.g., COPD, bronchiectasis, or severe asthma) patients and healthy individuals. It comprises ARGs that provide resistance to multiple antibiotic classes, including β-lactams (i.e., oxa-255, and *cfxA2*), macrolides (i.e., msrD, *ermB*, *ermX*, and *ermF*), aminoglycosides (i.e., aac(3)-VIIa and *aph(3)-IIIa*), tetracyclines (i.e., tetW, *tetA*, *tetB*, *tetD*, and *tetO*), diaminopyrimidine (i.e., dfrA1), fluoroquinolones (i.e., pmrA), lincosamides (i.e., lnuC), and chloramphenicol (i.e., catS) [3,4]. This core resistome challenges the paradigm of antibiotic exposure as the sole driver of resistance, evidenced by its presence even in antibiotic-naïve populations [6]. In addition, the ubiquitous presence of macrolide-resistance genes within the core resistome raises significant concerns for the long-term administration of macrolides in respiratory diseases [6,30].

Concerning the disease-specific resistome, distinctive ARG abundance and diversity patterns are evident among CRDs, with COPD and bronchiectasis patients presenting higher ARG levels than severe asthma patients and healthy individuals [30,75]. Intriguingly, recent antibiotic exposure does not consistently correlate with ARG prevalence, suggesting that the impact of antibiotic treatment on the resistome may be transient and warrants further longitudinal investigation [6,76]. Nonetheless, long-term antibiotic therapies aimed at preventing COPD exacerbations are associated with increased resistance to agents such as moxifloxacin, doxycycline, and azithromycin, highlighting the necessity of balancing short-term therapeutic gains against potential resistance development [30,77].

The application of clinical metagenomics in respiratory practice offers a powerful, integrative tool for profiling microbial taxonomy, function, and resistance patterns from a single specimen. Metagenomics is particularly valuable for precision respiratory medicine, allowing for tailoring therapeutic approaches based on a patient’s unique microbiota and resistome profile [6]. Despite these advancements, the resource-intensive nature of metagenomic analyses and the complexities of interpreting resistome data remain barriers to widespread adoption in clinical settings. As our understanding of the respiratory resistome evolves, it becomes increasingly clear that monitoring ARG dynamics, particularly in CRD management, is essential for developing sustainable and effective antibiotic stewardship strategies in respiratory medicine.

## 3. Therapeutic Strategies to Preserve Respiratory Microbiome Health and Combat Antibiotic Resistance

### 3.1. Antibiotic Stewardship Programs

The improper administration of antibiotics is one of the primary causes of the evolution of antibiotic-resistant microorganisms, prompting better approaches from healthcare personnel and patients. In this context, antibiotic stewardship programs (ASPs) offer a feasible strategy for encouraging responsible antibiotic consumption [78,79,80,81]. In recent years, ASPs have been implemented across ambulatory settings [78] and nursing homes [80]. Nonetheless, these complex programs aim equally at prescribers, patients, drug providers, policymakers, and the general public [79].

For instance, the Joint Commission in the United States outlined the requirements for ASPs, highlighting the importance of implementing ASPs in healthcare facilities to decrease antibiotic resistance and improve patient outcomes [81]. Regulatory and accreditation agencies have established (approved or proposed) that all hospitals should implement ASPs regardless of size. However, only the most intensive such interventions have been noted to significantly reduce the total and broad-spectrum antibiotic use compared to baseline [82].

Most evidence for the effectiveness of antibiotic stewardship (ABS) therapies is available at the hospital level. ABS in hospitals has demonstrated a favorable impact, with shorter hospital stays, reduced treatment duration without an increase in mortality, and decreased colonization and infection with resistant bacteria [79]. The evidence in favor of ASPs in the acute care setting is increasing. Yet, more helpful advice on staffing ratios and the resources required to implement these suggestions will improve the available information [81]. In addition, ABS implementation protocols face limitations in low- and middle-income countries, with only several initiatives reported as feasible at the international and local levels [79,81]. While significant progress has been made in implementing ASPs globally, addressing disparities in resources and adapting guidelines to diverse healthcare systems remains essential for their widespread effectiveness.

Early and accurate diagnosis of RT infections is crucial for properly managing patients [83]. To be significantly informative, the clinical laboratory should comprise both microbiological and virological methods [84]. RT infections diagnosis starts with a preliminary evaluation of the symptoms and signs, allowing the clinical microbiologist to determine the correct diagnostic workflow. However, the reliability of the diagnostic results is based on the collection, transport, storage, and processing of the respiratory sample specimen [85].

Traditionally, RT infection diagnostic workflow includes several tools for determining microbial and viral etiology, such as microscopic examination, conventional culture, antigen detection, and serology [84,85].

In diagnosing RT infections, coupling microscopy with respiratory samples’ Gram staining allows for the identification of polymorphonuclear cells and the presumptive recognition of bacterial morphology consistent with pathogens such as *Corynebacterium diphtheriae* and *Bordetella pertussis*, particularly in nasopharyngeal aspirates. However, Gram staining could not discriminate between streptococci causing pharyngotonsillitis or *N. meningitidis* and the nonpathogenic colonizers of the normal microbial population of the upper respiratory system [86]. Although it has several advantages, microscopy has limitations, including being laborious and time-consuming and not being recommended as a routine diagnostic of RT infections [84]. The gold standard for isolating and detecting respiratory pathogens remains bacterial culture. In the case of nasopharyngeal samples (e.g., swab, wash, aspirate, biopsy, scraping, or debridement), bacterial culture helps diagnose *B. pertussis*, *C. diphtheriae*, *Chlamydophila* spp., *N. meningitidis*, *S. aureus*, and *S. pyogenes* infections [86]. However, culture and susceptibilities are time-consuming, requiring several days for diagnosis and risking exposure across this time to expose patients to potentially ineffective therapies [87]. Serological tests are essential for identifying several atypical bacterial agents such as *B. pertussis*, *C. pneumoniae*, *M. pneumoniae*, and *Legionella* spp. However, the accurate clinical diagnosis is conditioned by the need for acute and convalescent sera to monitor seroconversion [84].

The advancements in new analytical approaches, such as molecular methods (nucleic acid amplification tests, metagenomics), allow researchers to broadly maximize the direct detection of respiratory pathogens, especially those hardly detectable and culturable [88,89].

Today, nucleic acid amplification tests, including standard, multiplex, and real-time polymerase chain reaction (PCR), are FDA-approved and could provide rapid, highly sensitive, and specific results in diagnosing RT infections [90]. Given the urgent need for prompt diagnosis, PCR assays are considered the new gold-standard diagnostic method for *B. pertussis* and SARS-CoV-2 infection [91]. The FDA-approved Unyvero PCR panel could detect *S. aureus*, *Acinetobacter baumannii*, *E. coli*, *H. influenzae*, *Klebsiella oxytoca*, *K. kneumoniae*, *K. variicola*, and *MM. catarrhalis*, *Morganella morganii*, *P. aeruginosa*, *Serratia marcescens*, *S. maltophilia*, *Legionella pneumophila*, *M. pneumoniae*, *Enterobacter cloacae complex*, *E. aerogenes*, *Proteus* spp., *Pneumocystis jirovecii*, *S. pneumoniae*, and *Citrobacter freundii* [92]. BIOFIRE FilmArray System (RT-PCR/nested multiplex PCR) Pneumonia Panel could detect 18 Gram-positive and Gram-negative bacteria, seven antibiotic resistance markers, nine viruses, and seven genetic markers of antibiotic resistance within one hour [93].

However, PCR assays have several limitations, including the acute contamination problem, the constant need to update their internal references for bacteria and viruses [90], and provide information only about the presence of the bacterial DNA, not the viability of the bacterial cell [94]. In contrast, metagenomic sequencing-based shotgun diagnostics do not use internal databases to compare the sample to known organisms or resistance patterns. Metagenomics involves the extraction of total DNA and/or RNA, fragmentation, library preparation, and depth sequencing, enabling pathogen genome assemblies and identifying new pathogen emergence [95]. Recently, a research team used metagenomic sequencing to investigate 205 clinical samples retrieved from 201 patients suspected of LRTIs. The results showed that metagenomic analysis has great potential in identifying microorganisms of clinical significance in LRTIs, with a rapid turnaround time [96].

### 3.2. Alternatives to Conventional Antibiotics

As conventional antibiotics continue to lose efficacy in front of MDR strains, alternative solutions must be sought. Several nontraditional antimicrobial agents have been explored concerning respiratory infections, aiming to combat the spread of pathogens and reduce the burden placed on patients and healthcare facilities. Recently, the combination of knowledge from physics, chemistry, microbiology, and nanotechnology, non-antibiotic approaches like antimicrobial peptides (AMPs), bacteriophages, and nanostructured materials have been proposed to circumvent the limitations of conventional drugs [66,97,98].

AMPs have gained increasing research interest for designing and synthesizing new therapeutic peptide analogs as they may present strong antimicrobial activity against antibiotic-resistant bacterial strains [99,100]. AMPs exhibit direct bactericidal properties and potent immunomodulatory functions, making them suitable candidates for controlling bacterial infections and treating chronic inflammatory diseases. Their mechanism of action supposes the destabilization of bacterial membranes, internalization, interaction with cellular organelles, and interference with cellular processes, effects that eventually build up toward destroying the bacteria. By suppressing inflammation, AMPs also help the organism achieve immune homeostasis, which supports bacterial clearance [99]. Moreover, given that AMPs possess a broad activity spectrum and do not interact with specific receptors, phenotypes resistant to these antimicrobials are rarely observed. Nonetheless, certain resistance cases have already been registered, including *S. aureus* resistance to dermcidin and reduced sensitivity of some bacteria to cationic AMPs [101]. Other associated drawbacks include AMPs’ modest toxicity [100] and the low yield and unwanted impurities of natural AMP isolation [101]. To overcome AMPs’ limitations, research has been directed toward exploring synthetic peptides and delivery systems. These approaches can help reduce associated toxicity, protect against degradation, improve targeting of airway infections, enhance antimicrobial and immunomodulatory properties, and diminish large-scale production costs [100,101].

Bacteriophages (also known as phages) represent another category of promising alternatives to traditional antibiotics. Bacteriophages are viruses that affect bacteria and are a potential solution for treating infections produced by MDR pathogens. These viruses are among Earth’s most abundant biological entities, are highly specific, and induce no effects in non-targeted bacteria. Compared to conventional drugs, bacteriophages have a “self-replicative” nature, which allows them to increase concentration at the infection site, reducing the necessity of repeated dose administration [97,102]. Moreover, phages can reach sites of infection in a manner that may not be possible through chemical compounds, persist for a longer period in the body, and exhibit low intrinsic toxicity, ensuring enhanced therapeutic outcomes [102]. Even though they can also induce bacterial resistance, bacteriophages coevolve with bacteria to overcome it. Notably, phage resistance may resensitize bacteria to antibiotics or render them avirulent [97]. In addition, phages were observed to augment the immune response by lysing bacterial cell walls, triggering immune activation. Hence, bacteriophage-based therapies eliminate bacteria directly and stimulate the immune system to combat infections [103]. However, several obstacles have been identified when utilizing phages in vivo, including loss of activity caused by external factors (e.g., exposure to conditions like organic solvents, temperature, pH, and salinity), risk of protein misfolding, aggregation, or denaturation, low bioavailability, low diffusion and penetration into tissues, and elimination by the host immune system. To avoid these issues, bacteriophages can be encapsulated in various delivery systems that protect their integrity and improve release at infection sites [97].

**Figure 5:**
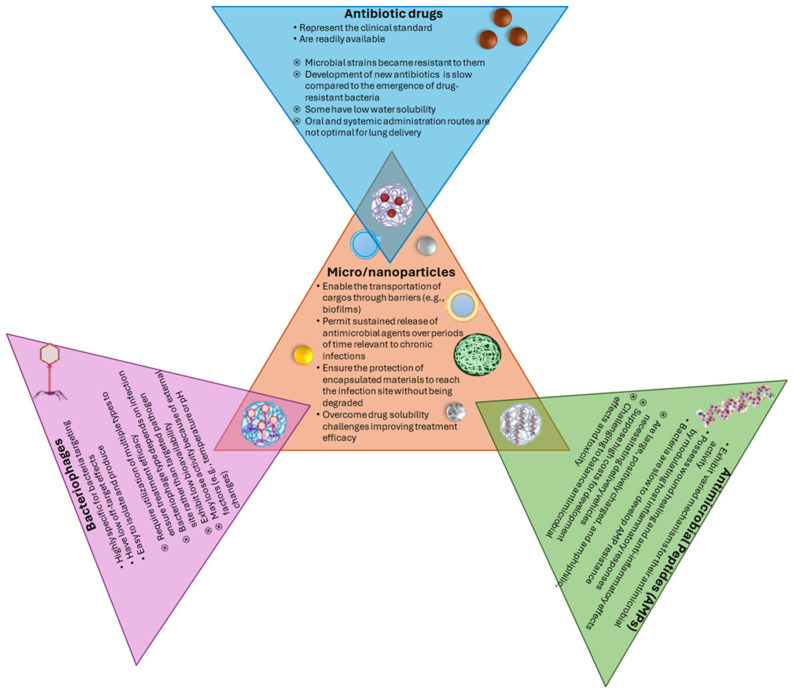
Overview of biomaterial strategies for lung infection treatment. Created based on information from [98,99,100,102].

In addition to these options, numerous studies have investigated the potential of various nanoparticles (NPs) for circumventing off-target toxicity and fighting against antibiotic-resistant infections of the respiratory tract, either as inherent antimicrobials or as efficient delivery vehicles [104,105]. Nanostructured systems based on metallic and metal-oxide NPs, carbon-based nanomaterials, lipid-based NPs, and polymeric NPs have been explored as carriers for improving the solubility of hydrophobic antimicrobial agents, ensuring sustained cargo release, prolonging systemic circulation time and antibiotic half-life, and lowering necessary drug doses [105]. Besides being paired with conventional antibiotics, nanomaterials can also encapsulate AMPs and bacteriophages, creating hybrid systems with synergistic properties (Figure 5).

Nanoparticle drug delivery systems are also involved in infection treatment due to their ability to ensure targeted antibiotic transport and release at the infection site, achieving the MIC with minimal side effects and reduced disruption of the host microbiota. The efficacy of nanocarriers can be enhanced through surface functionalization, incorporating stimuli-responsive ligands to improve cellular uptake, selectivity, payload capacity, and non-cytotoxicity [105,106]. Advanced “smart” systems releasing bacteriophages in response to specific triggers represent a particularly innovative approach to optimizing antimicrobial activity [102].

Active targeting in nanomedicine employs various biomolecules, including peptides, antibodies, nanobodies, proteins, nucleic acids, carbohydrates, and antimicrobial drugs, to enhance specificity against pathogens [106,107,108,109,110,111,112,113,114,115,116]. Such approaches allow the design of tailored nanostructures that interact with Gram-positive and Gram-negative bacteria’s distinct cell wall characteristics [117]. On one hand, Gram-negative bacteria feature an outer membrane with lipopolysaccharides and efflux pumps that confer resistance to antibiotics by limiting their entry or exporting them. On the other hand, Gram-positive bacteria lack this outer membrane but employ diverse mechanisms to counteract antibiotics. Additionally, mycobacteria, including *M. tuberculosis*, possess a unique cell envelope of mycolic acids, peptidoglycan, and arabinogalactan, creating a sturdy barrier to antibiotic penetration [3]. Nanostructures can offer customized and effective antimicrobial solutions by targeting these structural and functional bacterial features.

Concerning the base material of nanoparticulate delivery systems, polymers are often preferred due to their appealing physicochemical properties. Exhibiting great versatility, stability, good solubility, biocompatibility, biodegradability, and facile synthesis, various natural and synthetic polymeric NPs have been widely investigated for overcoming drug resistance and increasing the therapeutic activity of carried antimicrobial agents [61,118]. For example, Chen et al. [104] have proposed the utilization of poly(ethylene glycol)–poly(ε-caprolactone) (PECL) amphiphilic copolymers conjugated with vancomycin assembled as micelle carriers and loaded with ciprofloxacin. The delivery systems were tested on animal models, leading to higher survival rates in *P. aeruginosa*-infected mice, less bacterial colonization in lungs, and fewer alveolar injuries than free drugs, thus providing a feasible alternative for antibiotic release in response to the infection microenvironment. Differently, Walvekar and colleagues [119] reported on the encapsulation of vancomycin into hyaluronic acid-oleylamine (HA-OLA) conjugates that self-assembled as polymersomes. The developed nanocarriers worked in synergy with the carried drug, allowing for a stronger impact on the MRSA membrane, giving promise as a treatment for antibiotic-resistant infections.

Several recent studies have been conducted on chitosan-based nanosystems. For instance, Qiu et al. [120] associated this natural polymer with phosphatidylcholine and gentamicin to create antimicrobial NPs with enhanced activity. The researchers reported that the developed structures effectively destroy bacterial biofilms, kill intracellular bacteria, and impart negligible cytotoxicity. Ciro et al. [121] have alternatively proposed the use of ampicillin-loaded chitosan–polyanion nanoparticles, demonstrating their applicability, especially for treating *S. aureus* infections, with a two-fold increase in antimicrobial activity being noted in comparison to free ampicillin.

Interesting possibilities have also been registered when combining chitosan and alginate, as demonstrated by Kaur et al. [122]. The authors have used such combined nanoparticles as delivery vehicles for LysMR-5. The blank carriers possessed inherent antibacterial activity, while the addition of the cargo resulted in enhanced bactericidal effects against *S. aureus*. Differently, Scolari et al. [123] have co-loaded rifampicin and ascorbic acid into alginate–chitosan NPs. Tested against methicillin-sensitive *S. aureus* and MRSA, the developed nanosystem displayed a significant biocide activity attributed to the collaborative effects between the encapsulated antibiotics and polymeric carriers. In vivo studies revealed that the co-loaded NPs are suitable for antibiotic lung administration, being of interest for new treatment alternatives against pulmonary intracellular infections resistant to common medication.

On a different note, Falciani et al. [124] have proposed the utilization of single-chain dextran NPs as vehicles for delivering SET-M33, a branched synthetic AMP with demonstrated in vitro efficacy against approximately 100 Gram-negative multi-drug and extensively drug-resistant clinical isolates. This encapsulation system significantly enhanced lung residence time when administered via aerosol. It ensured high local doses of antimicrobials, showing promise for developing novel therapeutics, especially for treating recurrent infections.

Other highly investigated approaches concerning respiratory infections are lipid-based nanocarriers. Lipid NPs, such as solid lipid NPs and liposomes, offer desirable biocompatibility, stability, availability, non-toxicity, high loading capacity, protection of encapsulated drugs, improved interface interactions, controlled cargo release, and efficacy in reducing bacterial load [125,126,127]. Moreover, lipid-based NPs can inherently target infected macrophages through their unique features, ensuring precise delivery to the infection site and subsequently enhancing treatment outcomes [126]. For instance, Patil and Deshpande [128] demonstrated that loading clofazimine into nanostructured lipid carriers surface-functionalized with mannose allows for prolonged residence times in lung tissues. This effect has been attributed to the sustained drug release and the mannose receptor-mediated endocytosis, leading to a twofold greater bioavailability than free drug dispersion. Therefore, the researchers concluded that the developed nanosystems are a safe and effective solution for administering clofazimine via inhalation, marking a step forward in the management of tuberculosis disease.

Lipid-based NPs have also been extensively investigated as non-viral targeted carriers for nucleic acids due to their cationic nature at low pH, enabling lipid complexation with negatively charged RNA or DNA [129,130,131]. In this respect, studies have explored various lipid NPs to create mRNA-based vaccine formulations, envisaging novel solutions for managing infectious diseases [130]. The most known and widely used such nano-vaccines are Moderna and Pfizer/BioNTech formulations against severe acute respiratory syndrome coronavirus 2 (SARS-CoV-2). These lipid-based mRNA delivery systems were successful at a large scale, helping prevent severe symptoms and pathogen mutations during replication in the host’s cells throughout the global public health crisis [132,133,134,135].

Promising nanotechnology-based approaches also arise from utilizing a broad range of metal and metal oxide NPs. Nano-dimensional materials, such as silver [136,137,138,139], gold [140,141,142], iron oxide [143,144,145], zinc oxide [146,147,148], and titanium oxide [149,150,151,152], have been recognized for their intrinsic antimicrobial activities, being explored in numerous and varied formulations for fighting severe infections. For example, Pokhrel et al. [139] have designed a Smart Nano-Enabled Antiviral Therapeutic (SNAT) containing taxoid-decorated amino-functionalized positively charged silver NPs. The innovative SNAT was proven effective against SARS-CoV-2 infection, reducing the virus load in oral swabs and improving the lung health of tested animals (i.e., hamsters). Another anti-coronavirus nanoformulation was proposed by León-Gutiérrez et al. [152]. The authors tackled the benefits of TiO2 NPs functionalized with flavonoids to obtain a potent antiviral effect, interfere with the SARS-CoV-2 spike, and subsequently impair the cell fusion mechanism.

Alternatively, Celebi et al. [146] have recently reported on the synergistic activity of zinc oxide and zinc borate NPs, which have been effective against *K. pneumoniae* and *P. aeruginosa*. The combination of nanoparticles resulted in mixed antibacterial and cytotoxic effects, showing promise for treating lung cancer patients with diseases further complicated by pneumonia, which are otherwise challenging to manage with conventional antibiotics. Nonetheless, the authors acknowledge the limitations of their work, concluding that in-depth in vivo studies are further required to determine genotoxicity, excretion rates, and optimal NP administration methods. On another note, Souza et al. [140] have investigated the potential of polyethylene glycol (PEG)-capped gold NPs as delivery vehicles for AMP BP100, recognized for its low susceptibility to bacterial resistance. Through molecular simulations, the authors indicated the feasibility of these nanotherapeutics for dealing with infectious lung diseases, recommending future translation to in vitro and in vivo testing stages.

The composition and administration route of antimicrobial nanoformulations have been investigated in several studies. Research revealed that aerosol administration of nanoparticle-based therapeutics is a promising approach to enhancing the efficacy and precision of medication for respiratory infections. This route minimizes systemic exposure, reduces dosing frequency, and mitigates adverse effects such as the disruption of gut microbiota, ensuring that the cargo is directly delivered to the lungs [69,153]. Clinical validation of this strategy is exemplified by amikacin liposome inhalation suspension, the first inhalable antibiotic–nanomedicine approved by the US FDA [153]. Further innovations, such as incorporating mucoadhesive cationic groups into nanocarrier surfaces, were noted to increase lung retention time and improve pulmonary drug delivery.

Additionally, advanced designs enable controlled release in response to specific lung conditions, such as the acidic environment of inflamed tissues, or enhance targeting through electrostatic interactions with bacterial biofilms. Importantly, aerosolized antibiotics have demonstrated efficacy in reducing infection-related inflammation while supporting the immune microenvironment in the lung, and studies indicate they do not significantly promote antibiotic resistance, particularly in conditions like cystic fibrosis. Despite these advancements, future research must address the challenges of achieving uniform drug distribution and optimizing therapeutic outcomes for diverse pulmonary infections [31,69,98,153].

### 3.3. Probiotics and Microbiome Restoration

**Figure 6:**
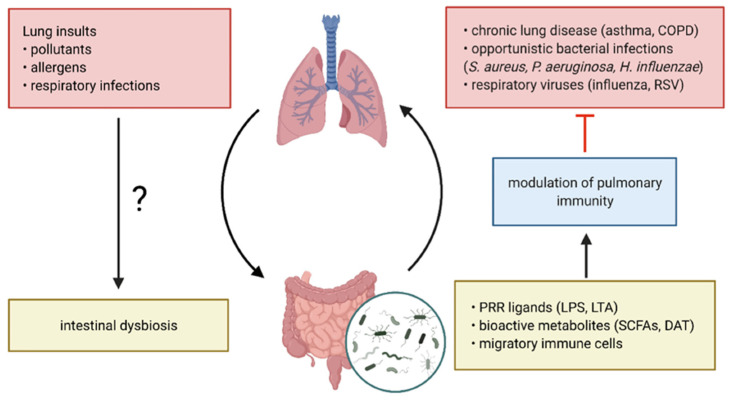
The gut–lung axis in respiratory disease—schematic representation of the bidirectional communication system. Reprinted from an open-access source [5]. Abbreviations: COPD—chronic obstructive pulmonary disease; DAT—desaminotyrosine; LPS—lipopolysaccharide; LTA—lipoteichoic acid; RSV—respiratory syncytial virus; SCFAs—short-chain fatty acids.

Not only are lungs no longer considered sterile, but the RTM has been noted to be impacted by microbial signals from distal body parts, such as the gut bacteria. This association has led to the discovery of the gut–lung axis [81,154,155]. This continuous bidirectional cross-talk between the gut and lung microbiotas is crucial in maintaining immune homeostasis and overall respiratory health [5,11,50] (Figure 6).

The gut–lung axis communication is facilitated by several mechanisms of action, including systemic dissemination of structural bacterial pattern recognition receptor (PRR) ligands (e.g., lipoteichoic acid and lipopolysaccharides), beneficial anti-inflammatory and antimicrobial metabolites (e.g., short-chain fatty acids—SCFAs, and desaminotyrosine—DAT), and migratory immune cells [5,11,155,156]. These interactions collectively shape pulmonary immunity, influencing susceptibility to respiratory pathogens and modulating chronic lung disease pathogenesis. Moreover, lung insults can also trigger intestinal dysbiosis, yet the underlying mechanisms have not been fully elucidated [5,50].

Dysbiosis in the gut (i.e., altered microbial diversity or composition) has been linked to inflammatory and infectious diseases, such as asthma, CF, COPD, tuberculosis, and lung cancer [50,154,156,157]. The reduced gut microbial diversity in the first month of life has been correlated with the development of childhood asthma [158,159]. Studies on the bacterial microbiota of children with advanced CF have also highlighted that higher gut microbial diversity correlates with prolonged periods of health and delayed disease exacerbation [158,160]. It has also been noted that, given the proximity of the abdominal and thoracic cavities, a gut–respiratory transmission of pathogens might occur, with pneumonia and peritonitis being frequently associated [11]. Moreover, studies performed on animal models of sinusitis revealed that sinus microbiota diversity modulates the pathogenicity of sinus-resident bacteria, emphasizing the role of microbial composition in health and disease [158]. In addition, gut dysbiosis during critical illnesses, such as COVID-19, suggests new perspectives on potential therapeutic avenues. Gastrointestinal symptoms in COVID-19 patients and the interaction of gut-respiratory microbial populations highlight the gut–lung axis as a target for future interventions [11]. The connections between the intestinal microbiome and respiratory diseases suggest the potential opportunities for novel approaches, including probiotic, prebiotic, and synbiotic interventions [154,160].

Probiotic supplementation holds promise for restoring microbial homeostasis and mitigating pulmonary diseases via its influence on the gut–lung axis. Administration of beneficial bacteria can reduce vulnerability to infections and manage LRT diseases, leveraging the cross-talk between intestinal and respiratory microbiota [5,20,161]. Supplementation with probiotics like *Lactobacillus* and *Bifidobacterium* strains has been proven effective in mouse models of influenza virus, RSV, and pneumonia, displaying antimicrobial, anti-inflammatory, and immunomodulatory activities. Dietary supplementation with *L. casei*, *L. rhamnosus*, or *Bifidobacterium longum* has been reported to enhance pulmonary pathogen clearance and reduce inflammation in experimental models of respiratory infection. Moreover, clinical studies and meta-analyses results indicate the modest role of probiotics in reducing the incidence and duration of URT infections, while regarding LRT, there is only some preliminary evidence of reducing the occurrence of ventilator-associated pneumonia (VAP) in high-risk patients [5].

Probiotics have also been explored in relation to viral respiratory infections, with particular focus on decreasing the risk of respiratory failure in COVID-19 patients [5]. For instance, supplementation with probiotics like *L. gasseri*, *L. rhamnosus*, *L. plantarum*, *B. bifidum*, and *B. subtilis* can inhibit viral protein, binding to host receptors and disrupting key viral replication processes. Probiotics can aid treatment by immune regulation, enhancing antigen presentation, and promoting balanced humoral and cellular responses to infection. In addition, the antioxidant capacity of probiotic bacteria is relevant and valuable in COVID-19 management, given the important role of redox homeostasis in curbing disease progression [20,161].

Interesting possibilities for restoring healthy respiratory microbiota can be borrowed from the methods applied in manipulating gut microbial communities. Particularly, it was noticed that a thoughtful alteration of the microbiota leads to colonization resistance to intestinal infections. For instance, fecal microbiota transplantation has been proven effective in treating conditions related to persistent *Clostridium difficile* infection and inflammatory bowel disease [69]. Based on such examples, microbial administration has also been explored for lung infections. It has been demonstrated that oral or intranasal inoculation with bacterial strains that can activate Nod2 receptors is beneficial in animal models. More specifically, mice were protected against respiratory infections by *S. aureus* or *K. pneumoniae*, with the effect depending on the strains’ capacity to produce cytokine GM-CSF [155].

With these aspects in mind, restoring a healthy microbiota holds promise as an intervention for pulmonary diseases characterized by imbalances in the microbial community. For example, this strategy can be applied in asthma, which is associated with disordered microbial communities and diminished commensal diversity. Similarly, restoring microbial balance has therapeutic potential for individuals whose microbiota has been compromised by long-term antibiotic use, as is the case with children with cystic fibrosis or primary ciliary dyskinesia, as well as adults with COPD [69,162]. Nonetheless, there are remaining challenges that have to be solved. The main limitation is that, unlike the gastrointestinal tract, the airways lack a readily transplantable source of microbiota from healthy individuals. Moreover, LRT commensal communities’ characteristics have not been fully elucidated, and the airways are frequently colonized by pulmonary pathogens that may not necessarily cause disease [69].

The hot topic of the gut–lung axis has reached the level of clinical trials, as evidenced by the series of interventional and observational studies recently registered on the Clinical.Trials.gov platforms (Table 3). The identified clinical trials emphasize the complex relationship between the gut and lung microbiota in the context of chronic and acute respiratory conditions, including COPD, post-COVID-19 syndrome, and bronchiectasis. Strategies like dietary modulation, probiotic supplementation, and fecal microbiota transplantation are under evaluation for improving immune response, reducing inflammation, and optimizing microbiota balance during or after infections. Despite varying completion dates and stages, these initiatives signify a promising frontier for translational research into precision medicine applications addressing both microbiota modulation and personalized therapies. As the results from these studies become publicly available, they are expected to deepen our understanding of microbiome-related mechanisms and open new avenues for integrative, microbiota-focused therapeutic strategies in respiratory medicine.

## 4. Future Research Directions

Emerging technologies for microbiome analysis are transforming our understanding of microbial ecosystems and their roles in health and disease. However, integrating data from bacterial and fungal microbiomes as well as viromes remains a critical frontier, as most current studies usually focus on one type of microorganism. Future research must focus on developing more sophisticated methodologies to analyze these microbiomes simultaneously, highlighting inter-microbial interactions and their effects on host physiology. Improved identification of strain-specific and species-specific functional traits and studying microbial metabolites in disease models can enable more precise diagnostic and therapeutic interventions [167]. For instance, the role of microbiomes in lung transplantation, lung cancer, and chronic respiratory diseases demonstrates how microbial balance disruptions influence immune responses and disease progression. Understanding these interactions could guide personalized therapeutic strategies, such as modulating the microbiome to prevent transplant rejection, allograft dysfunction, or lung cancer progression [168].

Precision medicine approaches are considered to revolutionize modern treatments, including those aimed at infections and associated microbiome-related conditions. Targeted therapies tailored to individual patient profiles, including pathogen identity, genotypic and phenotypic resistance, and host immune responses, could mitigate the collateral damage associated with broad-spectrum antimicrobial treatments [169]. Such strategies could concomitantly minimize disruptions to the microbiome while effectively eliminating pathogens, maintaining the delicate balance vital to combating AMR. Nevertheless, there are still challenges to implementing precision medicine, including the necessity of resource-intensive diagnostic methods, ethical considerations surrounding invasive procedures, and the need for extensive clinical validation. Nonetheless, evolution-informed treatment approaches that anticipate pathogen resistance mechanisms and preserve microbial balance hold promise for achieving sustainable therapeutic outcomes [63]. For example, integrating microbiome-based biomarkers into diagnostic frameworks can enable stratified treatment plans that enhance efficacy while minimizing unintended resistance proliferation [170].

Potential therapeutic targets are expanding, driven by insights into microbiome functionality and host–microbe interactions. Future studies may investigate targeting microbiome components such as bacterial efflux pumps in Gram-negative pathogens [3], fungal β-glucan pathways in lung cancer [168], or specific bacterial ligands in chronic respiratory diseases [52] as promising opportunities to disrupt disease progression. Emerging precision therapeutics, such as stimuli-responsive nanoparticles and phage therapies, offer innovative ways to target resistant pathogens selectively [102,105,106]. Moreover, exploring the complex connection between microbiomes at different body sites, such as gut–lung and oral–lung axes, may uncover previously unrecognized mechanisms of disease development, opening avenues for holistic therapeutic strategies [52,163,164,165,166].

## 5. Conclusions

The respiratory microbiome represents a critical yet underexplored reservoir of ARGs, which greatly impacts respiratory infection management. Respiratory tract microbiota and pathogenic microorganisms interact in a bidirectional manner, creating a delicate microbial balance that must be maintained to reduce the risk of diseases and antimicrobial resistance occurrence. Moreover, the gut–lung axis has been identified as a major regulatory pathway that connects intestinal microbiota with respiratory health and overall immune homeostasis.

Taking into account the underlying causes of antimicrobial resistance, the particular resistance mechanisms of respiratory pathogens, and the cross-talk between gut and lung microbiomes, innovative solutions for preventing and combating antibiotic-resistant respiratory infections can be envisaged. Comprehensive approaches encompassing antibiotic stewardship, microbiome restoration, and nanotechnology integration with AMPs, bacteriophages, and alternative therapeutic agents are essential for addressing the multifaceted challenges posed by respiratory tract infections and their resistance mechanisms and moving toward precision medicine.

Thus, as the era of microbiome research advances, translating these discoveries into clinical practice will necessitate multidisciplinary collaboration and rigorous validation to ensure safety and efficacy. Thus, through concerted efforts of scientists from different interconnected fields, the emerging solutions to antibiotic resistance can soon become impactful and sustainable healthcare strategies.

## Figures and Tables

**Table 1:** Terminology. Created based on information from [12,13,14,15,16,17,18,19,20].

Term	Definition
Microbiota	Living microorganisms coexisting in a defined habitat; a dynamic microbial community confined within a specified environment (e.g., human body and oral cavity) [12]
Microbiome	Collection of genomes from all the microorganisms in the environment, comprising the genetic information and inferred physicochemical properties of the gene products of a microbiota [13]
Metagenomics	A process of randomly sequencing the entire DNA present in a sample (including DNA from the host organism and microorganisms), which is further analyzed, organized, and identified by means of sequence databases and computational tools to highlight the genetic potential of the population [14]
Dysbiosis	Modification in microbiota composition linked to perturbation of local ecological conditions, usually linked with impaired host–microbe interactions [15]
Microdysbiosis	Ecosystem disturbance produced by the destruction of the microecological balance [16]
Antibiotic resistance	Ability of bacteria to develop defense mechanisms and evade the activity of antibiotic drugs [17]
Resistome	Assembly of quantity, identity, and functions of ARGs [18]
Prebiotics	Gut microbiota-accessible dietary fibers are able to nourish and promote beneficial bacteria growth [19]
Probiotics	Beneficial live microorganisms present in fermented foods and dietary supplements [20]

**Table 2:** Impact of antibiotics on the RTM in chronic respiratory diseases. Adapted from an open-access source [30].

Antibiotic	Disease	Analyzed Sample	Impact of Antibiotics on the Diversity of the Microbiome	Impact on the Relative Abundance of Bacterial Taxa
Oral macrolides	Bronchiectasis	Oropharyngeal swab	No impact on α-diversity measures	Difference between treated and placebo groups: ↓ *Actinomyces* and *Streptococcus* ↑ *Haemophilus* after 48 weeks of treatment
		Sputum	Increase in genus richness between baseline and 48 weeks in the treated group, but no difference with the placebo group	No change in the composition of the airway microbiome in the *P. aeruginosa*-dominated subgroup ↓ *H. influenzae* and ↑ *P. aeruginosa* in non-*P. aeruginosa*-dominated subgroup
	Severe asthma	Oropharyngeal swab	Impact on β-diversity measures	↓ *Fusobacteria* ↑ Firmicutes during treatment compared with the untreated group, but return to the pre-treatment state after a 1-month washout period
		Sputum	↓ Faith’s phylogenetic diversity	↓ Gammaproteobacteria (including *H. influenzae*) after 48 weeks of azithromycin treatment compared with a placebo group
	Moderate and severe asthma	Bronchoalveolar lavage of the right upper lung lobe	↓ Shannon’s diversity index	↓ *Prevotella*, *Staphylococcus,* and *Haemophilus* ↑ *Anaerococcus* between the pre- and post-treatment states
	Chronic obstructive pulmonary disease	Sputum	-	↓ of multiple taxa, mainly *Proteobacteria*
β-lactams	Cystic fibrosis	Nasal swabs	↑ Shannon’s diversity index	↓ *Moraxellaceae* ↑ other bacterial families (this increase was verified after more than one antibiotic treatment)
		Bronchoalveolar lavage, sputum, or deep throat swabs	↓ α-diversity between exacerbation and treatment ↓, α-diversity at therapeutic doses between baseline and treatment ↑ α-diversity at sub-therapeutic doses at the same time points	↓ *Haemophilus*, *Clostridiales,* and *Lachnospiraceae* ↑ *Fusobacterium* and *Pseudomonas* in the group treated at therapeutic doses between baseline and treatment samples No difference was observed in the sub-therapeutic group at the same time points No difference in the bacterial composition was observed in the two groups between post-recovery and baseline samples or between exacerbation and treatment samples
		Sputum	↓/↑ Shannon’s diversity index (depending on the type of treatment) ↓ Bray–Curtis β-diversity with ↑ AZLI cycles	↓ Relative abundance of some low-abundance taxa with antibiotic treatment: *Gemella*, two *Pasteurella* operational taxonomic units, two *Streptococcus* operational taxonomic units, *Oribacterium* and *Neisseria* ↓ *P. aeruginosa* ↑ anaerobes (*Prevotella* and *Veillonella*) in the first 72 h of treatment, but return to the baseline state after 8–10 days of treatment
Aminoglycosides	Cystic fibrosis	Sputum	↓ average species richness (Shannon and Simpson diversity indices) after 1 week of therapy. Return to baseline state after the end of TIP therapy No difference in Shannon’s diversity index	Most changes noticed between the baseline state and the first week of treatment occurred among low-abundance taxa, mostly facultative and obligate anaerobes (*Neisseria*, *Megasphaera*, *Granulicatella*, *Haemophilus*, *Streptococcus*, *Gemella*, *Rothia*, *Veillonella*, *Oribacterium*) ↓ *Parvimonas*
Association of different antibiotic classes in the treatment of cystic fibrosis exacerbation episodes	Cystic fibrosis	Sputum	↓ α-diversity (inverse Simpson index) ↑ Species richness through PEx and treatment periods, but returns to the baseline state during the recovery period	↓ *Prevotella melaninogenica* and *S. sanguinis* ↑ *Veillonella parvula* during the treatment period, but returns to the baseline state in post-recovery samples

↓, decrease; ↑, increase;

**Table 3:** Recent clinical studies investigating the gut–lung axis, as retrieved from ClinicalTrials.gov in December 2024.

ClinicalTrials.Gov Identifier	Official Title	Study Type	Intervention/ Treatment	Phase	(Estimated) Completion Date	References
NCT06271213	The Gut–Lung Axis and Respiratory Illness in Children	Observational	-	-	1 May 2028	-
NCT04813718	Post-COVID-19 Syndrome: A Pilot Study to Explore the Gut–Lung Axis	Interventional	Dietary Supplement: Omni-Biotic Pro Vi 5 Dietary Supplement: Placebo	N/A	31 December 2023	-
NCT03236480	Dynamic Changes in the Respiratory Microbiota and Its Relationship to Fecal Microbiota in Chronic Obstructive Pulmonary Disease	Observational	-	-	1 January 2019	[163]
NCT05623007	Dietary Modulation of Gut Microbiota on Nutritional Status and COVID-19 Infection in Adolescents: Gut–Lung Axis	Interventional	Dietary Supplement: Probiotics Behavioral: Counseling On Healthy Eating, Physical Activity, and Psychosocial Stimulation Dietary Supplement: Placebo Probiotics	2	2 November 2025	-
NCT05937815	Monitoring of the Intestine–Lung Axis of Cystic Fibrosis Patients Treated with the Combination Elexacaftor/Tezacaftor/Ivacaftor: Study of the Pulmonary and Gut Microbiota and Inflammation	Interventional	Procedure: Sample Collection	N/A	13 September 2024	-
NCT04490447	Identification of Microbiome and Metabolome of Bronchiectasis in Chinese Population and Role of the “Gut-lung Axis” in Chronic Respiratory Infection with *P. aeruginosa*.	Observational	-	-	1 September 2021	[164]
NCT04979065	Effect of Probiotic and Vitamin D Supplementation in Modulating Gut Dysbiosis, Nutrition, Inflammation, and Immune Status and Reduce Risk of COVID-19 in Obese People: Gut–Lung Axis Randomized Trial	Interventional	Dietary Supplement: Probiotics, Vitamin D Other: Placebo	N/A	30 December 2022	-
NCT03642548	A Prospective Multicenter Double-blind Randomized Clinical Trial of Probiotics Combined with Chemotherapy in the Treatment of Patients with Advanced Non-Small Cell Lung Cancer	Interventional	Drug: Bifico Drug: Placebo	3	1 March 2024	[165]
NCT05164445	Observational Participants Not Assigned to Intervention(s) Based on a Protocol, typically in the context of routine care	Interventional	Diagnostic Test: Transbronchial Forceps Biopsy Diagnostic Test: Transbronchial Forceps Biopsy + Transbronchial Cryobiopsy	N/A	31 August 2023	-
NCT04960878	The Effect of Synbiotics on the Upper Respiratory Tract Infection in Healthy Subjects: A Randomized Double-Blind Trial	Interventional	Dietary Supplement: Synbiotic Dietary Supplement: Placebo	N/A	5 January 2021	-
NCT04824222	Two-stage Study: Phase II/III—With a Pilot Safety Assessment in an Open-label Study and Phase III—a Multicenter, Randomized, Double-blind, Placebo-Controlled Evaluation of the Effect of Fecal Microbiota Transplantation as an Immunomodulation, in Addition to Standard Therapy, on the Risk Reduction in COVID-19 Disease Progression with Escalating Cytokine Storm and Inflammation	Interventional	Drug: Human Fecal Microbiota, MBiotix HBI Drug: Placebo Drug: SOC	3	December 2022	-
NCT04447144	Nutritional Habits: Do They Affect Coronavirus Disease 2019 (COVID-19) Infection Outcome? An Egyptian Experience	Observational	-	-	1 September 2020	[166]
NCT06348212	Effect of Probiotic Strain Lactobacillus Paracasei PS23 on Brain Fog in People with Long COVID	Interventional	Dietary Supplement: Lactobacillus Paracasei PS23 Dietary Supplement: Microcrystalline Cellulose	N/A	December 2025	-

N/A—not applicable.

## Data Availability

No new data were created or analyzed in this study.
